# Long-Term Longitudinal Evaluation of Six Commercial Immunoassays for the Detection of IgM and IgG Antibodies against SARS CoV-2

**DOI:** 10.3390/v13071244

**Published:** 2021-06-26

**Authors:** Iulia Nedelcu, Raluca Jipa, Roxana Vasilescu, Cristian Băicuș, Costin-Ioan Popescu, Eliza Manea, Laura E. Stoichițoiu, Larisa Pinte, Anca Damalan, Oana Simulescu, Irina Stoica, Madalina Stoica, Adriana Hristea

**Affiliations:** 1 Faculty of Medicine, University of Medicine and Pharmacy “Carol Davila” Bucharest, 050474 Bucharest, Romania; iulia-maria.nedelcu@drd.umfcd.ro (I.N.); cristian.baicus@umfcd.ro (C.B.); eliza.manea@drd.umfcd.ro (E.M.); larissa.0806@yahoo.com (L.P.); adriana.hristea@umfcd.ro (A.H.); 2 Department of Infectious Diseases Adults 4, National Institute for Infectious Diseases “Prof. Dr. Matei Bals”, 1 Dr. Calistrat Grozovici Street, 021105 Bucharest, Romania; scoicaru.anca@gmail.com; 3 Medlife Laboratory, 365 Calea Grivitei Street, 010719 Bucharest, Romania; roxanavasilescu62@gmail.com (R.V.); osimulescu@medlife.ro (O.S.); istoica@medlife.ro (I.S.); madalina.stoica@medlife.ro (M.S.); 4 Department of Internal Medicine, Colentina Clinical Hospital, 19-21 Stefan cel Mare Street, 72202 Bucharest, Romania; laura.elena.stoich@gmail.com; 5 Institute of Biochemistry of the Romanian Academy, 296 Splaiul Independentei, 060031 Bucharest, Romania; popescu80@hotmail.com

**Keywords:** COVID-19, SARS-CoV-2, serological assays, ELISA, CLIA, ECLIA, CMIA, IgG, IgM

## Abstract

The number of serological assays for SARS-CoV-2 has skyrocketed in the past year. Concerns have been raised regarding their performance characteristics, depending on the disease severity and the time of the analysis post-symptom onset (PSO). Thus, independent validations using an unbiased sample selection are required for meaningful serology data interpretation. We aimed to assess the clinical performance of six commercially available assays, the seroconversion, and the dynamics of the humoral response to SARS-CoV-2 infection. The study included 528 serum samples from 156 patients with follow-up visits up to six months PSO and 161 serum samples from healthy people. The IgG/total antibodies positive percentage increased and remained above 95% after six months when chemiluminescent immunoassay (CLIA) IgG antiS1/S2 and electro-chemiluminescent assay (ECLIA) total antiNP were used. At early time points PSO, chemiluminescent microparticle immunoassay (CMIA) IgM antiS achieved the best sensitivity. IgM and IgG appear simultaneously in most circumstances, and when performed in parallel the sensitivity increases. The severe and the moderate clinical forms were significantly associated with higher seropositivity percentage and antibody levels. High specificity was found in all evaluated assays, but the sensitivity was variable depending on the time PSO, severity of disease, detection method and targeted antigen.

## 1. Introduction

Since the WHO declared the COVID-19 outbreak a pandemic, an increasing amount of data regarding the immune response in SARS-CoV-2 infection has become available. However, the immunity remains incompletely elucidated; therefore, research on this subject is ongoing and evolving [1]. Serological assays might be relevant not only for epidemiological and vaccine studies but also for the management of the patients presenting with late complications of the disease, when RT-PCR may be falsely negative and externally validated SARS-CoV-2 serologic assays are needed.

The currently available serological assays are variable regarding the format: enzyme-linked immunosorbent assay (ELISA), chemiluminescent immunoassay (CLIA), gold immunochromatographic assay (GICA), lateral flow immunoassay (LFIA), and the most recent, multiplex immunoblot assay that is able to evaluate antibody responses to various SARS-CoV-2 proteins at the same time. The viral antigens used (nucleocapsid protein (NP), subunits of the spike (S) glycoprotein, the spike glycoprotein receptor binding domain (RBD)) and the antibody detected (immunoglobulin class M (IgM), immunoglobulin class G (IgG), or both) are also different among various assays available [2,3]. Serological assays typically detect antibodies against S glycoprotein and/or NP, since these are the most immunogenic proteins of SARS-CoV-2 [4].

The antibody detection rates across different serological tests are variable, depending, among other factors, on the timing of seroconversion [5]. It is also important to assess the diagnostic performance when it comes to the COVID-19 disease severity, which may vary from a mild, to a critical form [6]. In addition, a potential problem is the cross-reactivity of the antibodies to non-SARS-CoV-2 coronavirus proteins.

A large number of serologic assays are commercially available, and significant concern remains with respect to their performance characteristics considering the limited experience with these new assays. Therefore, independent validations before their broad use in routine clinical practice is needed [2]. According to the Foundation for Innovative New Diagnostics (FIND), a “global non-profit organization driving innovation in the development and delivery of diagnostics”, there are about 200 commercial serological tests in COVID-19 diagnostics, with this disease currently having the most serological tests available, more than any other known infectious disease [7].

We aimed to assess the clinical performance and agreement between six commercially available assays based on CLIA, electro-chemiluminescent assay (ECLIA), chemiluminescent microparticle immunoassay (CMIA), and ELISA. These methods were used to assess IgG, IgM, and total antibodies against SARS-CoV-2 in COVID-19 patients. We evaluated the sensitivity of the assays in accordance with the symptoms’ onset and the severity of the disease. Seroconversion and dynamics of antibodies in response to SARS-CoV-2 infection were also analyzed.

## 2. Materials and Methods

### 2.1. Patients

We performed a prospective multicenter study conducted in two hospitals, both from Bucharest, Romania: the National Institute of Infectious Diseases “Prof. Dr. Matei Bals” and Colentina Clinical Hospital. The study was approved by the local ethics committee of the two hospitals.

In order to assess the sensitivity, we collected sera from adult patients with COVID-19 confirmed by at least two positive reverse transcription polymerase chain reaction tests (RT-PCR) from nasopharyngeal swabs.

We collected 528 serum samples from 156 patients, each of whom reported the day of the symptoms’ onset. From 128 patients admitted for evaluation, we aimed to collect serum samples obtained at admission, and at follow-up visits on day 7, 14, 28, and 84. We also used serum samples from 28 patients from whom we had only one sample with known time post-symptom onset (PSO). Specimens were stored at 4 °C for up to 5 days. They were then centrifuged, and serum samples were aliquoted and frozen at −20 °C until analysis/before being processed. Informed consent was obtained from all included patients. We recorded demographic characteristics (age and gender), the date of symptoms onset, the clinical form of the disease (mild, moderate severe/critical) according to the WHO criteria, and the comorbidities [8].

The specificity was evaluated using serum samples as negative controls, collected in October 2019 from 161 healthy people within a seroprevalence study, upon the consent of the investigators in this study.

### 2.2. Assays

In order to confirm SARS-CoV-2 infection, nasopharyngeal swabs were collected on viral transport media (VTM) (Copan, Brescia, Italy), and these were further tested for SARS-CoV-2 RNA using Allplex™ SARS-CoV-2 Assay (Seegene, Seoul, Korea) and CFX96 Real-Time PCR Detection System (Bio-Rad, Hercules, CA, USA). We tested serum samples for SARS-CoV-2-specific antibodies using six commercially available assays: Elecsys^®^ anti-SARS-CoV-2 assay (Roche, Basel, Switzerland), LIAISON^®^ SARS-CoV-2 S1/S2 IgG (DiaSorin, Saluggia, Italy), SARS-CoV-2 IgG and SARS-CoV-2 IgM (Abbott, Chicago, IL, USA), and ELISA kits KT-1032 EDI™ IgG and KT-1033 EDI™ IgM (Epitope, San Diego, CA, USA). Three assays targeted the nucleocapsid protein (Abbott IgG, Roche total antibody and Epitope IgG and IgM) and two had S protein-based targets (DiaSorin and Abbott IgM).

The Elecsys^®^ anti-SARS-CoV-2 assay is an automated electrochemiuminescent assay (ECLIA) using a recombinant protein representing the N protein of SARS-CoV-2. This is a sandwich immunoassay whose output is a cutoff index (COI) used in a qualitative manner to determine whether a sample is positive or negative for antibodies. According to the manufacturer, a COI < 1 is considered “negative” or “not detected”.

The LIAISON^®^ SARS-CoV-2 S1/S2 IgG uses an indirect CLIA technology for the quantitative determination of IgG antibodies against specific recombinant S1 and S2 antigens. The analyzer automatically calculates SARS-CoV-2 S1/S2 IgG antibody concentrations expressed as arbitrary units (AU/mL); sample results should be interpreted as positive if AU/mL ≥ 15.0, equivocal if 12.0 ≤ AU/mL < 15.0, and negative if AU/mL < 12.0.

SARS-CoV-2 IgG (Abbott) is a chemiluminescent microparticle immunoassay (CMIA) used for the qualitative detection of IgG antibodies to the N protein of SARS-CoV-2. The cutoff is 1.4 index (S/C). SARS-CoV-2 IgM (Abbott) assay is designed to detect IgM antibodies that bind the S RBD antigen of SARS-CoV-2. The cutoff is 1.00 index (S/C).

The ELISA kits KT-1032 EDI™ IgG and KT-1033 EDI™ IgM are designed for the qualitative measurement of the human anti-COVID-19 IgG and IgM antibody against NP in serum, using a microplate-based enzyme immunoassay technique. The cutoffs were calculated for each working series using the formula PCO = 1.1 × (m_NC_ + 0.18) for positive cutoff and NCO = 0.9 × (m_NC_ + 0.18) for negative cutoff. Measured values ≤ NCO were interpreted as negative, measured values ≥ PCO were interpreted as positive, and measured values >NCO but <PCO were interpreted as borderline or equivocal.

To assess the specificity, we used serum samples collected in September 2019 for a seroprevalence study from healthy adult people. A SARS-CoV-2-specific antibody search was performed according to the manufacturers’ instructions at Medlife Laboratory using different automated platforms as follows: Architect i2000 for SARS-CoV-2 IgG and SARS-CoV-2 IgM (Abbott), Cobas e411 for Elecsys^®^ anti-SARS-CoV-2 assay (Roche), Liaison XL for LIAISON^®^ SARS-CoV-2 S1/S2 IgG, and Dynex DSX for ELISA kits KT-1032 EDI™ IgG and KT-1033 EDI™ IgM (Epitope).

### 2.3. Analysis

All statistical analyses were conducted using IBM^®^ SPSS^®^ Statistics 26.0. We used descriptive statistics to analyze the data. Quantitative variables were expressed as median and compared using Student’s t-test. A two-sided *p*-value <0.05 was considered statistically significant.

We treated equivocal results as “positive”. Thirty-eight results were equivocal with ELISA IgG (Epitope), 21 with ELISA IgM (Epitope), one with CMIA IgG (Abbott), one with SARS-CoV-2 IgM (Abbott), and 19 with IgG antiS1/S2 (DiaSorin). We used MedCalc software v19.8 to calculate the sensitivity and the specificity. Percentage of agreement and kappa index were calculated with GraphPad Prism software v5.0 (San Diego, CA, USA).

## 3. Results

The study group consisted of 156 patients, 59.6% male, with a median age of 50.5 (IQR 40–59) years. The time PSO varied between one and 212 days, median time PSO of 16. The concomitant medical conditions, such as type 2 diabetes mellitus, obesity, cardiovascular diseases, and immunosuppression conditions are shown in Table 1.

**Figure 1:**
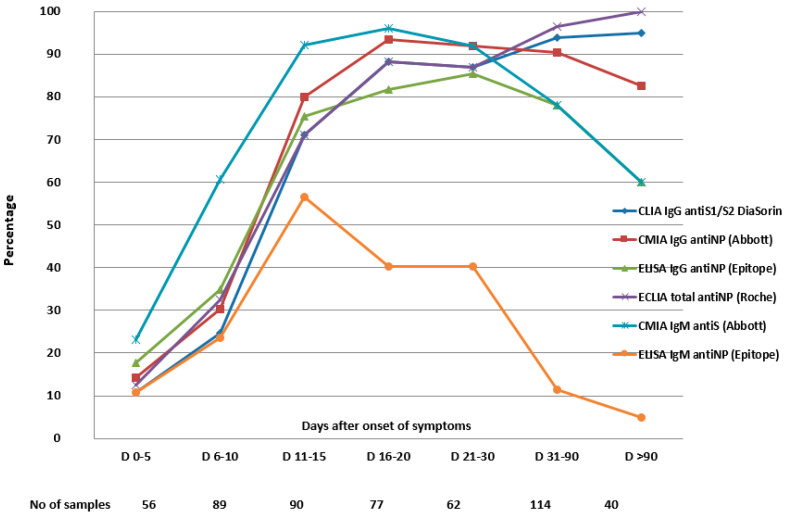
Dynamic trends of seropositivity rate according to the evaluated assays. Each time point from the x-axis represents the cumulated seropositivity rate at that specific time point.

We collected from each patient a mean number of 3.3 samples (range 1–5). The clinical sensitivity varied with the time PSO, as it is shown in Table 2 and Figure 1.

### 3.1. Sensitivity Dynamics and Specificity of the Assays Detecting IgG Antibodies

The overall sensitivity of the assays testing for IgG/total antibodies varied between 64.9% with ELISA IgG antiNP, (Epitope) and 70.4% with CMIA IgG antiNP (Abbott) as well as with ECLIA total antiNP (Roche) (Table 2).

The rate of IgG seropositivity increased over time, but some patients did not develop IgG antibodies, and the proportion was different among the assays. Moreover, we assessed the rate of seroconversion in 128 patients, from whom we collected serial serum samples. These results are shown in Table 3. 

In the interval 11–15 days, 71.1% samples tested by CLIA IgG antiS1/S2 (DiaSorin), 75.5% by ELISA IgG antiNP (Epitope), and 80% by CMIA IgG anti NP (Abbott) became positive. 

The rate of IgG or total antibodies positivity increased and remained at more than 95% for antibodies detected by CLIA IgG antiS1/S2 (DiaSorin) and ECLIA total antiNP (Roche), for sera collected beyond three months. Nevertheless, the rate of positivity of antiNP IgG antibodies detected by CMIA IgG (Abbott) decreased slightly from 91% for sera collected at three weeks to 82% after three months, and the positivity rate of IgG antibodies detected by ELISA decreased from 85% at three weeks to 60% after three months PSO (Table 2, Figure 1).

In addition to the 40 patients followed more than three months PSO and tested by all six assays, there were 31 patients tested by ECLIA total antiNP (Roche) and CLIA IgG antiS1/S2 (DiaSorin), for which serum samples were available for long-term surveillance, from 91 up to 271 days (median duration of 103 days [IQR 92–213]). The sensitivity was 100% using ECLIA total antiNP (Roche), and 97.2% using CLIA IgG antiS1/S2 (DiaSorin) (Table 2).

There were 21 (13.4%), 8 (5.1%), and 1 (0.6%) patients assessed by ELISA IgG antiNP (Epitope), CMIA IgG antiNP (Abbott), and CLIA IgG antiS1/S2 (DiaSorin) for which the result became seronegative. The median time from symptom onset to the first seronegative result after seroconversion was: 90 (IQR 86.5–94.5), 90.5 (IQR 86.5–93.7), and 46 days for ELISA IgG antiNP (Epitope), CMIA IgG antiNP (Abbott), and CLIA IgG antiS1/S2 (DiaSorin), respectively. All positive serum samples tested by ECLIA total antiNP (Roche) remained positive.

The specificity was assessed testing 161 controls by CLIA IgG antiS1/S2, CMIA IgG antiNP, ELISA IgG antiNP, and 158 controls by ECLIA total antiNP. The specificity was more than 97% for all targeted antibodies (Table 2).

### 3.2. The Sensitivity Dynamics and the Specificity of the Assays Detecting IgM Antibodies

The sensitivity of CMIA IgM antiS (Abbott) was 74.6%, while the overall sensitivity of ELISA IgM antiNP (Epitope) was 28.2%. The percentage of positive antibodies by ELISA (Epitope) was 56.6% at 11–15 days and had an important drop after four weeks PSO (Table 2).

Out of 128 patients for whom we collected serial serum samples, 102 (79.6%) had seroconversion by CMIA IgM antiS (Abbott) and 70 (54.6%) by ELISA IgM antiNP (Epitope). These results, along with the median time of IgM seroconversion are shown in Table 3.

The IgM antibodies were not detected by ELISA IgM antiNP (Epitope) in 44 (34.3%) patients and by CMIA IgM antiS (Abbott) in 3 (2.3%) patients (Table 3). In addition, there were 42 (32.8%) samples tested by ELISA IgM antiNP (Epitope) and 27 (21.1%) samples tested by CMIA IgM antiS (Abbott), which become seronegative at 31.5 days (IQR 22–49.7) and 88 days (IQR 71–93) PSO.

The sensitivity of combined IgM and IgG (positive if any of these was positive) is included in Table 2 and is higher than detecting either IgM or IgG antibodies.

We assessed the order in which IgM and IgG appeared by CMIA IgM antiS and CMIA IgG antiNP (Abbott) in 82 patients for which the samples from visits at 7, 14, and 28 days, in addition to the inclusion sample, were available (Table 4).

The specificity of CMIA IgM antiS and ELISA IgM antiNP was assessed testing 161 controls and was more than 97% for all targeted antibodies.

### 3.3. The Agreement between the Assays

The highest agreement had the assays detecting IgG antibodies against NP; 78% for CMIA IgG antiNP (Abbott) and ELISA IgG antiNP (Epitope), and 77.3% between CMIA IgG antiNP (Abbott) and ECLIA total antiNP (Roche). Between the assay detecting IgG antiS1/S1 antibodies (DiaSorin) and the assay detecting antibodies against NP, the agreement was lower, but still substantial, 71.1% for CMIA IgG antiNP (Abbott) and 70% for ECLIA total antibodies (Roche). We found moderate agreement between ELISA IgG (Epitope) as well as ECLIA total antibodies (Roche) on one hand (62.4%), and CLIA IgG antiS1/S2 (DiaSorin), on the other hand (62%). The 95% CIs are shown in the Table 5. The lowest agreement of (19%) was between the assays detecting IgM antibodies, CMIA IgM antiS, and ELISA IgM antiNP.

### 3.4. Antibody Dynamics According to the Disease Severity

**Figure 2:**
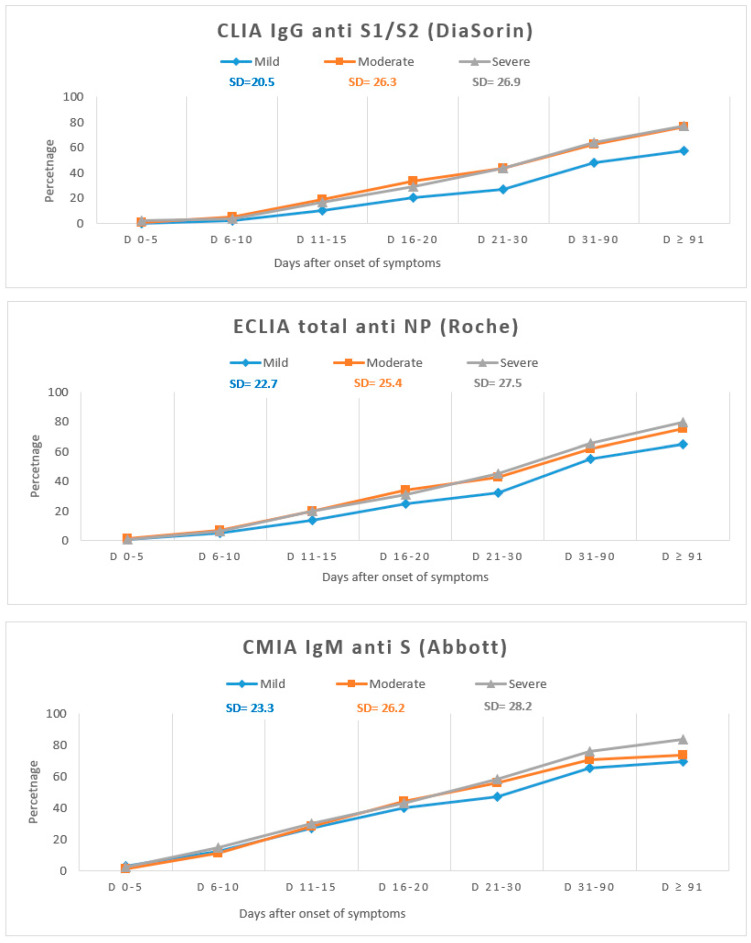

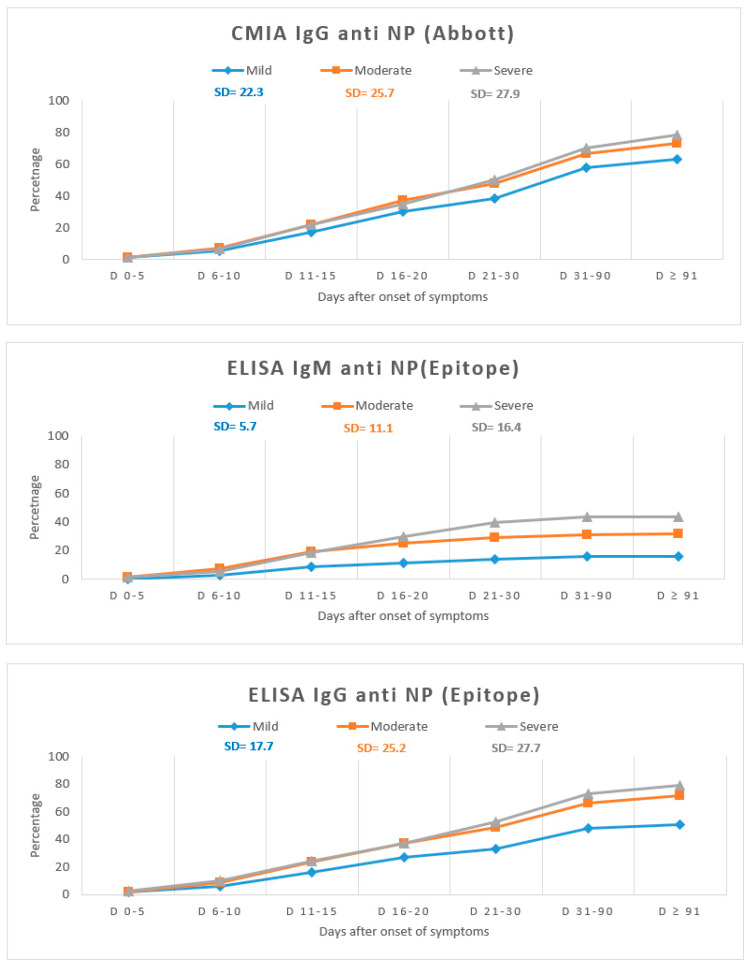
Variation in antibodies positivity by disease severity. SD = standard deviation. Each time point from the x-axis represents the cumulated seropositivity rate at that specific time point.

Since the clinical form of the disease and the moment when the sera were obtained have an effect upon the assay performance, we calculated for each test the antibody positivity at different time points PSO (Table S1). The variation in antibody positivity with the disease severity is shown in Figure 2. Overall, IgG and IgM antibodies appeared earlier in moderate and severe forms, when compared with mild forms of the disease. 

On the other hand, ELISA (Epitope) had the poorest performance in detecting antibodies in patients with mild forms.

Until day 10 there was no difference between the assays; after 90 days, for all the assays, there were significant differences in detecting antibodies according to the disease form, with higher rate of seropositivity in severe forms (Table S2). In between the aforementioned periods of time, ELISA IgM and IgG antiNP (Epitope) had the highest rate of significant differences in detecting antibodies according to the severity of the disease, while CMIA IgM antiS (Abbott) showed no significant difference.

Only one of the six assays (CLIA IgG antiS1/S2-Diasorin) is intended for the quantitative detection of SARS-CoV-2 antibodies. The antibody concentration detected by CLIA IgG antiS1/S2 according to the disease severity and the time PSO is shown in Table 6.

The median antibody level assessed by a quantitative method CLIA IgG antiS1/S2 was significantly higher (*p* < 0.001) in patients with severe disease (240 AU/mL), than in patients with moderate (88.9 AU/mL) or mild disease (59.9 AU/mL) after three months.

## 4. Discussion

In concordance with previous results, we found that most patients, but not all, develop anti-SARS-CoV-2 antibodies, while the frequency and the dynamic of the seroconversion are variable, depending on the studied population, the time from symptoms onset, and also on the assays, detecting antibodies against different targets (antiS and antiNP), by different methods (CMIA, ELISA, etc.) [9,10,11].

In some studies, the IgG positivity was consistently higher than the IgM-positive rate [5,12]. It was also the case in our study for IgM antibodies detected by ELISA (Epitope), but not for IgM detected by CMIA (Abbott). We found that IgM antibodies detected by CMIA (Abbott) had the highest positivity rate at <5 days (23%), and this trend was maintained for up to three weeks before the positivity rate began to decrease. Moreover, when performed during the first 10 days after symptoms onset, the sensitivity was 83.8%. It was suggested that IgM antibodies should not be used to diagnose acute infections, since they appear almost simultaneously with the IgG antibodies [13].

We found that most of our samples became positive simultaneously for both IgM and IgG antibodies, but in one-third of the patients, IgM antibodies appeared before the IgG antibodies. Our results indicate that SARS-CoV-2 IgM antiS response might be detected early in the course of the disease, having a potential role in the diagnosis of acute infection, in symptomatic patients presenting outside the window of positivity for RT-PCR-based SARS-CoV-2 tests. In addition, we found a sensitivity for CMIA IgM (Abbott) of 96% at three weeks, and the IgM antibodies were detected in 60% of a subgroup of the 40 patients followed after three months, in contrast with other study in which IgM antibodies became undetectable by the same method by day 37 [14].

IgG antibodies against NP and IgM against S have been found to appear almost in tandem, with similar median times to seroconversion of 11.0 and 10.8 days, respectively [15]. In our study, some samples were positive, both for IgM and IgG antibodies, at 2–3 days PSO. Nevertheless, the median time to earliest detected IgG antibodies was 11–13 days, similar with the median of 12 days in 16 studies including 4348 patients [16]. For IgM antibodies we found the median time to the earliest detected antibodies of 10–11 days, which was slightly higher than 7 days in 12 studies including 1715 patients in the previously mentioned systematic review [16].

The sensitivity was higher if either IgM or IgG antibodies were detected positive versus IgM or IgG alone, and this approach may provide better results in clinical practice [3,17].

It was suggested that the detection of antibodies against NP might be associated with a decrease in the time to seroconversion in human coronavirus infections [4] and also that the detection of the N protein is more sensitive than the detection of the S1 for SARS-CoV-2 [18]. Other studies that evaluated homemade ELISAs for antiN and antiS antibodies did not find a difference in regard to the percentage of positive samples at different intervals between the assays detecting antibodies against S1/S2 and against NP [19]. We found that IgM antibodies detected by CMIA IgM antiS appeared faster than ELISA IgM antiNP and faster than IgG antibodies. IgG antibodies occurred faster with ELISA anti NP (Epitope), CMIA antiNP, (Abbott), and ECLIA total antibodies antiNP (Roche), than with the antiS assay (DiaSorin). The interassay variability might be explained, at least in part, by the differences in the nature and the structure of the target, but the cutoff might also play a role [20]. It is not clear why the assays targeting the same antigen have differences in performance over time [21].

Comparable with the observations noted by other groups, we found differences among the commercial serologic assays that we evaluated, according to the time PSO. Compared with other antibody classes, IgG lasts longer. We noticed that the IgM-positive rate showed a trend to decline after four weeks; however, the IgG-positive rate increased, and then became stable over time, except for the IgG antibodies detected by ELISA, which also dropped after four weeks PSO.

Three weeks after the symptoms’ onset, the sensitivity of ELISA IgG was similar with the sensitivity reported by Whitman et al. for the same assay, ranging from 38.3% (at 1–5 days) to 90.9% (at >20 days). Contrarily, the sensitivity of ELISA IgM in our study was much lower (from 10% at 1–5 days to 40% at >20 days) than that reported by Whitman et al. in which the sensitivity varied from 17.9% at 1–5 days to 81.8% at >20 days [22]. The differences between our findings and the results of Whitman et al., who analyzed moderate and severe hospitalized patients, might be related to the sample size and patients’ characteristics, since we included 221 samples from 62 (39.7%) patients with mild disease.

In our study, the IgG assays positivity at more than 15 days (85.6%–93.1%) was lower than the positivity rate reported by the manufacturers (95%–100%). The apparent discrepancy may be most likely explained by the low IgG titer of samples in the selection. Indeed, Lagerqvist et al. showed that the sensitivities of different serology assays decrease for low IgG titer samples. This is the case for samples from early post symptom onset, in the late convalescent phase, in asymptomatic or mild infections, which are largely represented in our selection [23].

The sensitivities at 16–20 days by CMIA IgG antiNP (Abbott), CLIA IgG antiS1/S2 (DiaSorin), and ECLIA total antiNP (Roche) (88.3%–93.5%) were similar to the sensitivity of 88.2% reported at 15–21 days in a Cochrane review of SARS-CoV-2 antibody testing, which included 54 cohort studies with 15,976 samples, published in November 2020. Likewise, the sensitivity of CMIA IgM antiS (Abbott) was similar in the first week with the sensitivity reported in this systematic review (23%), but at 16–21 days, was better in our study (96.1% versus 75.4%) [24]. After four weeks, the sensitivity of the assays detecting IgM decreased across multiple studies, including ours.

Another study evaluating ECLIA total antiNP (Roche) and CLIA IgG antiS1/S2 (DiaSorin) found higher sensitivities than in our study after two weeks from symptom onset (92% and 88%, respectively) but comparable sensitivities after 30 days after symptom onset (100% and 97.5%, respectively) [25].

The overall sensitivity in our study was also similar with the results of a study in which the authors determined the antibody response against SARS-CoV-2 proteins using CMIA antiNP IgG (Abbott), CLIA IgG antiS1/S2 (DiaSorin), and ECLIA total antiNP (Roche), and found the sensitivity of 70.9%, 63.2% and 71.8%, respectively. Nevertheless, with all three assays, the 100% sensitivity was reached three weeks after the positive RT-PCR. This study analyzed the antibody response by reporting their length after the positive RT-PCR and not the duration of symptoms; besides, the subjects were hospitalized patients, most probably severe patients, with one-third of them requiring admission in an intensive care unit [26]. Moreover, in another study, the reported positivity rates by CMIA antiNP IgG (Abbott), CLIA IgG antiS1/S2 (DiaSorin), and ECLIA total antiNP (Roche) were 92.7%, 95.7%, and 96.8%, respectively at >14 days [27]. A possible explanation for the lower sensitivity found in our study could be the high percentage of patients with mild forms of COVID-19 who are known to develop fewer antibodies.

There are studies with an extended follow-up of the antibody response (up to five months), with CMIA antiNP IgG, which showed a significant decrease in sensitivity, as we found in our study [21,28,29]. However, the antibody response against NP antigen using ECLIA total antiNP antibodies was found to be persistent in several studies, similar with our findings [28,29]. A hypothesis for the sustained antibody response with an assay detecting total antibodies (ECLIA, Roche) compared with IgG assays is that an additional response of non-IgG antibodies isotypes may appear [20].

Studies with a longer follow-up are, however, still scarce in the literature. For the samples that we collected between 90 and 271 days, we found a similar sensitivity with the ones reported in two studies that used multiple commercial assays (including ECLIA total antiNP antibodies) for the evaluation of antibodies’ long-term persistence. In one study, 58 persons with asymptomatic or mildly symptomatic SARS-CoV-2 infection were followed-up to 8 months, and in another study, 84 individuals were followed-up to 10 months; the seropositivities were 90% and 94%, respectively [20,30]. Likewise, antibodies detected by CLIA IgG antiS1/S2 (DiaSorin) persisted in 95% of our study population.

For public health policies, the durability of the humoral immune response following the symptoms’ onset is still a matter of debate of major importance. Thus, robust serological assays with long-term high sensitivity post infection are needed. Sensitive qualitative tests may be useful for seroprevalence studies in population. Our study indicates that ECLIA total antiNP Roche anti-SARS-CoV-2 assay for the qualitative detection of anti-SARS-CoV-2 NP antibodies might be used for this purpose, for at least six months post-disease. A long-term evaluation of the sensitivity of different assays, may help prioritize the serology tests that should be used to determine the correlates of protection either after SARS-CoV-2 infection or after vaccination. Thus, sensitive and quantitative determination of antiS IgG antibodies will be required to monitor the protection status. We evaluated only one method for antiS IgG determination and it seems suitable to monitor antiS IgG levels for at least six months. Our study underscores the importance of evaluating multiple assays in an unbiased sample selection before concluding on the durability of the immune response. Thus, antiNP and antiS antibodies are present at least six months after symptoms onset. Our study showed that ELISA IgG antiNP (Epitopes) is not appropriate for serological evaluation at late time points PSO due to decreased sensitivity. The drop in sensitivity six months PSO is due most likely to the high number of samples from patients with mild form of disease, as it was recently reported [31]. In order to have a better view of the immune status of the COVID-19 convalescent patients, the binding assays should be complemented by sera neutralization assays, which are currently performed for our cohort. We will perform further investigations, including a neutralization test, which might assist a better understanding of the antibodies’ dynamics, as well as the discrepancies between the assays.

The specificity was very good for all assays, for all targeted antibodies, with narrow confidence intervals. However, we did not include in our study sera from patients with different infections, but only from healthy people. Prior studies on the prevalence of antibodies against commonly circulating coronaviruses showed that over 90% of individuals over the age of 50 have specific antibodies, potentially responsible for cross reactivity [32]. However, not only the accuracy of a test itself, but also the pre-test probability, which can vary widely, should be considered when interpreting the serological test results [33]. Considering both sensitivity and specificity, ECLIA total antiNP (Roche) and CLIA IgG antiS1/S2 (DiaSorin) had the best diagnostic performance after one month PSO.

Overall, more studies found that the sensitivity, the antibody levels, and earlier seroconversion are positively associated with the disease severity [6,9,11,34,35]. Our results agreed with the data from a review published by Mackey et al., which found in 17/25 studies an association between the disease severity and higher levels of antibodies [16]. We found that the disease severity was associated with an earlier seroconversion in IgG antibodies, detected by all methods targeting IgG.

Maximum follow-up in other studies published until March 2021 was up to 120 days [16]. Data beyond three months of follow-up are limited, and the long-term follow-up (with 71 samples collected at more than 90 days) is one of the strengths of our study, together with a large sample size, including more than 200 samples from patients with mild disease. So far, sensitivity has mostly been evaluated in hospitalized patients with moderate and severe forms, and it was unclear whether tests are able to detect lower antibody levels, mainly associated with mild or asymptomatic forms.

Like many other studies, we included only patients with COVID-19 confirmed by a RT-PCR. Nevertheless, COVID-19-positive cases who are RT-PCR-negative should be included in order to assess the utility of serological tests in clinical practice. Regarding the specificity, we did not include in our study sera from patients with different infections but only those from healthy people. However, probably, most of these patients had common viral infections (including coronavirus) during the three months before blood sampling; therefore, they may have developed antibodies against these infections, which did not interfere with the studied assays for SARS-CoV-2 (rate of false positives less than 3%).

## 5. Conclusions

In summary, the assays evaluated have a high specificity, but the sensitivity is variable, depending not only on the time of symptoms onset and the severity of illness, but also on the method and the antigen targeted. Therefore, the results of serological testing should be carefully interpreted in the context of the clinical findings. Nevertheless, testing for combined IgM and IgG might improve their performance, including in acute infection, but more important, in patients with late presentation of COVID-19, atypical symptoms, or prolonged symptoms.

## Figures and Tables

**Table 1:** Clinical characteristics and demographics of patients included in the study.

Characteristic COVID-19 Study Population (*n* = 156)	
Age, median IQR)	50.5 (40–59)
Male sex N (%)	93 (59.6)
**Chronic Medical Condition**	
Hypertension N (%)	49 (31.4)
Congestive heart failure N (%)	6 (3.8)
Coronary artery disease N (%)	12 (7.6)
Prior stroke N (%)	6 (3.8)
Type 2 diabetes mellitus (%)	25 (16)
Obesity N (%)	22 (14.1)
Cancer (solid or hematological)	8 (5.1)
Chronic liver disease N (%)	7 (4.4)
Chronic kidney disease N (%)	3 (1.9)
Asthma or other chronic respiratory diseases N (%)	7 (4.4)
Systemic inflammatory diseases N (%)	1 (0.6)
Other immunosuppression conditions N (%)	2 (1.2)
**Duration of Symptoms at Inclusion in the Study, Median (IQR)**	
Disease severity	16 (10–22)
Mild N (%)	62 (39.7%)
Moderate N (%)	53 (33.9%)
Severe N (%)	41 (26.2%)

**Table 2:** Clinical sensitivity according to the time post symptom onset.

	Overall Sensitivity N, % (95% CI)	D 0–5 N, % (95% CI)	D 6–10 % (95% CI)	D 11–15 % (95% CI)	D 16–20 % (95% CI)	D 21–30 % (95% CI)	D 31–90 % (95% CI)	D ≥ 91 % (95% CI)	Specificity % (95% CI)
Sample No.	528	56	89	90	77	62	114	40	161
**CMIA (Abbott)**									
IgM antiS	394 74.6 (70.6–78.2)	13 23.2 (12.9–36.4)	54 60.6 (49.7–70.8)	83 92.2 (84.6–96.8)	74 96.1 (89–99.1)	57 91.9 (82.1–97.3)	89 78 (69.3–85.2)	24 60 (43.3–75.1)	2 98,7 (95.5–99.8)
IgG antiNP	372 70.4 (66.3–74.3)	8 14.2 (6.3–26.2)	27 30.3 (21–40.9)	72 80 (70.2–87.6)	72 93.5 (85.4–97.8)	57 91.9 (82,1–97.3)	103 90.3 (83.3–95)	33 82.5 (67.2–92.6)	2 98,7 (95.5–99.8)
IgM/IgG *	437 82.7 (79.2–85.8)	13 23.2 (12.9–36.4)	56 62.9 (52–72.9)	86 95.5 (89–98.7)	75 97.4 (90.9–99.6)	60 96.7 (88.8–99.6)	111 97.3 (92.5–99.4)	36 90 (76.3–97.2)	4 97.5 (93.7–99.3)
**ELISA (Epitope)**									
IgM antiNP	149 28.2 (24.4–32.2)	6 10.7 (4–21.8)	21 23.5 (15.2–33.7)	51 56.6 (45.8–67)	31 40.2 (29.2–52)	25 40.3 (28.5–53.5)	13 11.4 (6.21–18.87)	2 5 (0.6–16.9)	4 97.5 (93.7–99.3)
IgG antiNP	343 64.9 (60.7–69)	10 17.8 (8.9–30.4)	31 34.8 (25–45.6)	68 75.5 (65.3–84)	65 81.8 (71.3–89.6)	54 85.4 (74.2–93.1)	90 78 (69.3–85.2)	25 60 (43.3–75.1)	3 98.1 (94.6–99.6)
IgM/IgG *	359 68 (63.8–72)	10 17.9 (8.9–30.4)	33 37.1 (27.1–48)	73 81.1 (71.5–88.6)	66 85.7 (75.9–93)	58 93.5 (84.3–98.2)	92 80.7 (72.2–87.5)	27 67.5 (50.9–81.4)	5 96.2 (92–98.6)
**Sample No.**	**559**	**56**	**89**	**90**	**77**	**62**	**114**	**71**	**161**
CLIA IgG antiS1/S2 DiaSorin	390 69.8 (65.7–73.5)	6 10.7 (4–21.8)	22 24.7 (16.1–35)	64 71.1 (60.6–80.1)	68 88.3 (78.9–94.5)	54 87 (76.1–94.2)	107 93.8 (87.7–97.5)	69 97.2 (83–99.3)	2 98.7 (95.5–99.8)
**Sample No.**	**559**	**56**	**89**	**90**	**77**	**62**	**114**	**71**	**158**
ECLIA total antiNP Roche	403 70.4 (66.3–74.3)	7 12.5 (5.1–24)	29 32.5 (23–43.3)	64 71.1 (60.6–80.1)	68 88.3 (78.9–94.5)	54 87 (76.1–94.2)	110 96.4 (91.2–99)	71 100 (91.1–100)	0 100 (97.7–100)

* Positive if either IgM or IgG is positive.

**Table 3:** Seroconversion rate in patients with serial serum samples.

	CLIA IgG antiS1/S2 (DiaSorin)	CMIA IgG antiNP (Abbott)	ELISA IgG antiNP (Epitope)	ECLIA Total antiNP (Roche)	CMIA IgM antiS (Abbott)	ELISA IgM antiNP (Epitope)
IgG/IgM persistently negative N * (%)	3 (2.3)	4 (3.1)	9 (7.0)	2 (1.5)	3 (2.3)	44 (34.3)
Patients with seroconversion N * (%)	112 (87.5)	110 (85.9)	107 (83.6)	108 (84.3)	102 (79.6)	70 (54.6)
IgG positive patients at inclusion N * (%)	13 (10.1)	14 (10.9)	12 (9.3)	18 (14.0)	13 (10.1)	14 (10.9)
Median time (min, max) PSO until seroconversion (days)	13 (2.36)	13 (2.35)	11 (2.30)	13 (3.36)	10 (2.22)	11.5 (3.32)

* N = number of patients.

**Table 4:** The order in which IgM and IgG antibodies appeared.

Simultaneous seroconversion N (%)	47 (57.3%)	IgM and IgG positive at inclusion N (%)	4 (4.9%)
IgM before IgG N (%)	23 (28.0%)	IgM positive but IgG persistently negative N (%)	4 (4.9%)
IgG before IgM N (%)	2 (2.4%)	IgG positive but IgM persistently negative N (%)	2 (2.4%)

**Table 5:** Agreement between the evaluated assays (528 samples) (95% confidence interval).

	ELISA IgG antiNP (Epitope) k, SE of k (95% CI)	CLIA IgG antiS1/S2 (DiaSorin) k, SE of k, (95% CI)	CMIA IgG antiNP (Abbott) k, SE of k, (95% CI)	CMIA IgM antiS (Abbott) k, SE of k (95% CI)
ECLIA total antiNP **(Roche)**	0.624, 0.036 (0.55–0.69) **moderate**	0.697, 0.034, (0.63–0.76) **substantial**	0.773, 0.030 (0.71–0.83) **substantial**	
ELISA IgG antiNP **(Epitope)**		0.622, 0.036, (0.55–0.69) **moderate**	0.780, 0.029 (0.72–0.83) **substantial**	
CLIA IgG antiS1/S2 **(DiaSorin)**			0.706, 0.034, (0.64–0.77) **substantial**	
ELISA IgM antiNP **(Epitope)**				0.192, 0.024, (0.14–0.23) **fair**

95% CI = 95% confidence interval. k = Cohen’s kappa. SE of k = standard error for kappa statistic.

**Table 6:** Median antibody concentration detected by CLIA IgG antiS1/S2 according to the disease severity and time PSO *.

	D 0–10		D 11–15		D 16–20		D 21–30		D 31–90		D ≥ 91		TOTAL	
Disease severity	N	Median (AU/mL) [IQR]	N	Median (AU/mL) [IQR]	N	Median (AU/mL) [IQR]	N	Median (AU/mL) [IQR]	N	Median (AU/mL) [IQR]	N	Median (AU/mL) [IQR]	N	Median (AU/mL) [IQR]
**Mild**	66	3.8 [3.8–13]	36	11.6 [3.8–88.6]	33	32.4 [7.8–108.7]	21	31.8 [4.6–114.4]	57	61.4 [10.7–112.8]	25	65.2 [15.9–157.6]	237	21.1 [4.3–64.3]
**Moderate**	48	3.8 [3.8–51.6]	35	30.8 [3.8–163]	31	60.5 [13.9–165.4]	24	72.3 [12.8–139.3]	41	102 [52.9–206]	29	131.5 [25.1–511.9]	208	54.4 [13.9– 105]
**Severe**	31	3.8 [3.8–55.5]	20	79 [10.6–156]	16	109.5 [25.3-223.9]	20	135 [49.8–246.1]	28	170 [98.2–340]	19	202 [91.7–806]	134	108 [21.2–177.5]
**TOTAL**	145	3.8 [3.8–30.2]	91	29.2 [3.8–121.6]	80	55 [11.5–152]	65	76.6 [8–186.4]	126	92.2 [22.3–220]	73	96.9 [20.5–462.2]	581	44.6 [7.8–104]

* *p* < 0.001. *p* calculated using Kruskal–Wallis test.

## Data Availability

For data supporting reported results please contact the correspondent author.

## Supplementary Materials

The following are available online at https://www.mdpi.com/article/10.3390/v13071244/s1, Table S1: Samples used to assess the variation in antibody kinetics (timing and duration) by disease severity.

## References

[1] Acuti Martellucci C., Flacco M.E., Cappadona R., Bravi F., Mantovani L., Manzoli L. (2020). SARS-CoV-2 pandemic: An overview. Adv. Biol. Regul..

[2] Theel E.S., Harring J., Hilgart H., Granger D. (2020). Performance Characteristics of Four High-Throughput Immunoassays for Detection of IgG Antibodies against SARS-CoV-2. J. Clin. Microbiol..

[3] Shah J., Liu S., Potula H.H., Bhargava P., Cruz I., Force D., Bazerbashi A., Ramasamy R. (2021). IgG and IgM antibody formation to spike and nucleocapsid proteins in COVID-19 characterized by multiplex immunoblot assays. BMC Infect. Dis..

[4] Meyer B., Drosten C., Müller M.A. (2014). Serological assays for emerging coronaviruses: Challenges and pitfalls. Virus Res..

[5] Zhang J.J.Y., Lee K.S., Ong C.W., Chan M.Y., Ang L.W., Leo Y.S., Chen M.I., Lye D.C.B., Young B.E. (2021). Diagnostic performance of COVID-19 serological assays during early infection: A systematic review and meta-analysis of 11 516 samples. Influenza Respir. Viruses.

[6] Garcia-Beltran W.F., Lam E.C., Astudillo M.G., Yang D., Miller T.E., Feldman J., Hauser B.M., Caradonna T.M., Clayton K.L., Nitido A.D. (2021). COVID-19-neutralizing antibodies predict disease severity and survival. Cell.

[7] Venter M., Richter K. (2020). Towards effective diagnostic assays for COVID-19: A review. J. Clin. Pathol..

[8] World Health Organization Coronavirus Disease (COVID19) Pandemic. https://www.who.int/emergencies/diseases/novel-coronavirus-2019.

[9] Zhao J., Yuan Q., Wang H., Liu W., Liao X., Su Y., Wang X., Yuan J., Li T., Li J. (2020). Antibody Responses to SARS-CoV-2 in Patients with Novel Coronavirus Disease 2019. Clin. Infect. Dis..

[10] Wolfel R., Corman V.M., Guggemos W., Seilmaier M., Zange S., Müller M.A., Niemeyer D., Jones T.C., Vollmar P., Rothe C. (2020). Virological assessment of hospitalized patients with COVID-2019. Nature.

[11] Long Q.X., Liu B.Z., Deng H.J., Wu G.C., Deng K., Chen Y.K., Liao P., Qiu J.F., Lin Y., Cai X.F. (2020). Antibody responses to SARS-CoV-2 in patients with COVID-19. Nat. Med..

[12] Jin Y., Wang M., Zuo Z., Fan C., Ye F., Cai Z., Wang Y., Cui H., Pan K., Xu A. (2020). Diagnostic value and dynamic variance of serum antibody in coronavirus disease 2019. Int. J. Infect. Dis..

[13] Hanson K.E., Caliendo A.M., Arias C.A., Englund J.A., Hayden M.K., Lee M.J., Loeb M., Patel R., Altayar O., El Alayli A. (2020). Infectious Diseases Society of America Guidelines on the Diagnosis of COVID-19: Serologic Testing.

[14] Orner E.P., Rodgers M.A., Hock K., Tang M.S., Taylor R., Gardiner M., Olivo A., Fox A., Prostko J., Cloherty G. (2021). Comparison of SARS-CoV-2 IgM and IgG seroconversion profiles among hospitalized patients in two US cities. Diagn. Microbiol. Infect. Dis..

[15] Ng D.L., Goldgof G.M., Shy B.R., Levine A.G., Balcerek J., Bapat S.P., Prostko J., Rodgers M., Coller K., Pearce S. (2020). SARS-CoV-2 seroprevalence and neutralizing activity in donor and patient blood from the San Francisco Bay Area. Nat. Commun..

[16] Mackey K., Arkhipova-Jenkins I., Armstrong C., Gean E., Anderson J., Paynter R.A., Helfand M. (2021). Antibody Response Following SARS-CoV-2 Infection and Implications for Immunity: A Rapid Living Review.

[17] Risch M., Weber M., Thiel S., Grossmann K., Wohlwend N., Lung T., Hillmann D., Ritzler M., Ferrara F., Bigler S. (2020). Temporal Course of SARS-CoV-2 Antibody Positivity in Patients with COVID-19 following the First Clinical Presentation. BioMed Res. Int..

[18] Brochot E., Demey B., Touzé A., Belouzard S., Dubuisson J., Schmit J.L., Duverlie G., Francois C., Castelain S., Helle F. (2020). Anti-spike; Anti-nucleocapsid and Neutralizing Antibodies in SARS-CoV-2 Inpatients and Asymptomatic Individuals. Front. Microbiol..

[19] To K.K., Tsang O.T., Leung W.S., Tam A.R., Wu T.C., Lung D.C., Yip C.C., Cai J.P., Chan J.M., Chik T.S. (2020). Temporal profiles of viral load in posterior oropharyngeal saliva samples and serum antibody responses during infection by SARS-CoV-2: An observational cohort study. Lancet Infect. Dis..

[20] Favresse J., Eucher C., Elsen M., Gillot C., Van Eeckhoudt S., Dogné J.M., Douxfils J. (2021). Persistence of Anti-SARS-CoV-2 Antibodies Depends on the Analytical Kit: A Report for Up to 10 Months after Infection. Microorganisms.

[21] Muecksch F., Wise H., Batchelor B., Squires M., Semple E., Richardson C., McGuire J., Clearly S., Furrie E., Neil G. (2021). Longitudinal analysis of clinical serology assay performance and neutralising antibody levels in COVID19 convalescents. J. Infect. Dis..

[22] Whitman J.D., Hiatt J., Mowery C.T., Shy B.R., Yu R., Yamamoto T.N., Rathore U., Goldgof G.M., Whitty C., Woo J.M. (2020). Evaluation of SARS-CoV-2 serology assays reveals a range of test performance. Nat. Biotechnol..

[23] Lagerqvist N., Maleki K.T., Verner-Carlsson J., Olausson M., Dillner J., Wigren Byström J., Monsen T., Eriksson J., Bogdanovic G., Muschiol S. (2021). Evaluation of 11 SARS-CoV-2 antibody tests by using samples from patients with defined IgG antibody titers. Sci. Rep..

[24] Deeks J.J., Dinnes J., Takwoingi Y., Davenport C., Spijker R., Taylor-Phillips S., Adriano A., Beese S., Dretzke J., Ferrante di Ruffano L. (2020). Antibody tests for identification of current and past infection with SARS-CoV-2. Cochrane Database Syst. Rev..

[25] Favresse J., Cadrobbi J., Eucher C., Elsen M., Laffineur K., Dogné J.M., Douxfils J. (2020). Clinical performance of three fully automated anti-SARS-CoV-2 immunoassays targeting the nucleocapsid or spike proteins. J. Med. Virol..

[26] Van Elslande J., Decru B., Jonckheere S., Van Wijngaerden E., Houben E., Vandecandelaere P., Indevuyst C., Depypere M., Desmet S., André E. (2020). Antibody response against SARS-CoV-2 spike protein and nucleoprotein evaluated by four automated immunoassays and three ELISAs. Clin. Microbiol Infect..

[27] National SARS-CoV-2 Serology Assay Evaluation Group (2020). Performance characteristics of five immunoassays for SARS-CoV-2: A head-to-head benchmark comparison. Lancet Infect. Dis..

[28] Gudbjartsson D.F., Norddahl G.L., Melsted P., Gunnarsdottir K., Holm H., Eythorsson E., Arnthorsson A.O., Helgason D., Bjarnadottir K., Ingvarsson R.F. (2020). Humoral Immune Response to SARS-CoV-2 in Iceland. N. Engl. J. Med..

[29] Sun B., Feng Y., Mo X., Zheng P., Wang Q., Li P., Peng P., Liu X., Chen Z., Huang H. (2020). Kinetics of SARS-CoV-2 specific IgM and IgG responses in COVID-19 patients. Emerg. Microbes Infect..

[30] Choe P.G., Kim K.H., Kang C.K., Suh H.J., Kang E., Lee S.Y., Kim N.J., Yi J., Park W.B., Oh M.D. (2021). Antibody Responses 8 Months after Asymptomatic or Mild SARS-CoV-2 Infection. Emerg. Infect. Dis..

[31] Peluso M.J., Takahashi S., Hakim J., Kelly J.D., Torres L., Iyer N.S., Turcios K., Janson O., Munter S.E., Thanh C. (2021). SARS-CoV-2 antibody magnitude and detectability are driven by disease severity, timing, and assay. medRxiv.

[32] Gorse G.J., Patel G.B., Vitale J.N., O’Connor T.Z. (2010). Prevalence of antibodies to four human coronaviruses is lower in nasal secretions than in serum. Clin. Vaccine Immunol..

[33] Watson J., Richter A., Deeks J. (2020). Testing for SARS-CoV-2 antibodies. BMJ..

[34] Hu W.T., Howell J.C., Ozturk T., Benameur K., Bassit L.C., Ramonell R., Cashman K.S., Pirmohammed S., Roback J.D., Marconi V.C. (2020). Antibody Profiles According to Mild or Severe SARS-CoV-2 Infection, Atlanta, Georgia, USA. Emerg. Infect. Dis..

[35] Young B.E., Ong S.W.X., Ng L.F.P., Anderson D.E., Chia W.N., Chia P.Y., Ang L.W., Mak T.M., Kalimuddin S., Chai L.Y.A. (2020). Singapore 2019 Novel Coronavirus Outbreak Research team. Viral dynamics and immune correlates of COVID-19 disease severity. Clin. Infect. Dis..

