# Supplementary material for: Long-Term Longitudinal Evaluation of Six Commercial Immunoassays for the Detection of IgM and IgG Antibodies against SARS CoV-2
Source: Viruses. 2021 Jun 26;13(7):1244. doi: 10.3390/v13071244 (PMC8310110; doi:10.3390/v13071244)
Supplement: Supplementary file 1 [file viruses-13-01244-s001.zip › viruses-1250721-supplementary.pdf]

			Intervals of time post symptoms-onset (days)						
	**Disease severity**	**Number of samples**	**D 0-5**<br>**N (%)**	**D 6-10**<br>**N (%)**	**D 11-15**<br>**N (%)**	**D 16-20**<br>**N (%)**	**D 21-30**<br>**N (%)**	**D 31-90**<br>**N (%)**	**D ≥ 91**<br>**N (%)**
CLIA IgG antiS1/S2 DiaSorin	Mild	236	1 (0.4)	6 (2.5)	18 (7.6)	25 (10.5)	16 (6.7)	49 (20.7)	23 (9.7)
	Moderate	203	2 (0.9)	11 (5.4)	28 (13.7)	30 (14.7)	20 (9.8)	39 (19.2)	28 (13.7)
	Severe	133	3 (2.2)	5 (3.7)	18 (13.5)	16 (12)	19 (14.2)	27 (20.3)	18 (13.5)
CMIA IgG anti NP Abbott	Mild	221	3 (1.3)	10 (4.5)	26 (11.7)	28 (12.6)	18 (8.1)	44 (19.9)	11 (4.9)
	Moderate	184	3 (1.6)	11 (5.9)	27 (14.6)	28 (15.2)	20 (10.8)	34 (18.4)	12 (6.5)
	Severe	123	2 (1.6)	6 (4.8)	19 (15.4)	16 (13)	19 (15.4)	25 (20.3)	10 (8.1)
ELISA IgG anti NP Epitope	Mild	221	4 (1.8)	9 (4)	23 (10.4)	23 (10.4)	14 (6.3)	33 (14.9)	6 (2.7)
	Moderate	184	3 (1.6)	13 (7)	27 (14.6)	26 (14.1)	21 (11.4)	32 (17.3)	11 (5.9)
	Severe	123	3 (2.4)	9 (7.3)	18 (14.6)	16 (13)	19 (15.4)	25 (20.3)	8 (6.5)
ECLIAtotal anti NP Roche	Mild	236	3 (1.2)	9 (3.8)	21 (8.8)	27 (11.4)	17 (7.2)	54 (22.8)	24 (10.1)
	Moderate	203	3 (1.4)	12 (5.9)	26 (12.8)	29 (14.2)	18 (8.8)	38 (18.7)	28 (13.7)
	Severe	133	1 (0.7)	8 (6)	18 (13.5)	15 (11.2)	19 (14.2)	27 (20.3)	19 (14.2)
CMIA IgM anti NP Abbott	Mild	221	7 (3.1)	21 (9.5)	32 (14.4)	29 (13.1)	16 (7.2)	40 (18.1)	9 (4)
	Moderate	184	3 (1.6)	18 (9.7)	32 (17.3)	29 (15.7)	22 (11.9)	27 (14.6)	6 (3.2)
	Severe	123	3 (2.4)	15 (12.2)	19 (15.4)	16 (13)	19 (15.4)	22 (17.8)	9 (7.3)
ELISA IgM anti NP Epitope	Mild	221	1 (0.4)	5 (2.2)	13 (5.8)	6 (2.7)	6 (2.7)	4 (1.8)	1 (0.4)
	Moderate	184	3 (1.6)	11 (5.9)	22 (11.9)	11 (5.9)	7 (3.8)	4 (2.1)	1 (0.5)
	Severe	123	2 (1.6)	5 (4)	16 (13)	14 (11.3)	12 (9.7)	5 (4)	0 (0)

Table S1: Samples used to assess the variation in antibodies kinetics (timing and duration) by disease severity

	**Day 0-5**	**Day 0-10**	**Day 0-15**	**Day 0-20**	**Day 0-30**	**Day 0-90**	**Day >90**
CLIA IgG antiS1/S2 **DiaSorin**	0.25	0.19	0.01	0.11	<0.01	<0.01	<0.01
CMIA IgG anti NP **Abbott**	0.96	0.78	0.44	0.30	0.047	0.047	<0.01
ELISA IgG anti NP **Epitope**	0.87	0.36	0.10	0.03	<0.01	<0.01	<0.01
ECLIA total anti NP **Roche**	0.83	0.59	0.15	0.10	0.016	0.10	<0.01
CMIA IgM anti NP **Abbott**	0.10	0.70	0.83	0.67	0.07	0.10	0.01
ELISA IgM anti NP **Epitope**	0.45	0.07	<0.01	<0.01	<0.01	<0.01	<0.01

Table S2 – p values for the assays seropositivity according to the severity of the disease and for specific time points
